# LONG-TERM POVERTY, SPATIAL DISADVANTAGE, AND MULTIPLE EXCLUSIONS IN LATER LIFE: A CASE IN SHANGHAI

**DOI:** 10.1093/geroni/igz038.1402

**Published:** 2019-11-08

**Authors:** Hongmei Tong, Daniel, W L Lai, Lun Li

**Affiliations:** 1 MacEwan University, Edmonton, Alberta, Canada; 2 The Hong Kong Polytechnic University, Hong Kong, Hong Kong; 3 University of Calgary, Calgary, Canada

## Abstract

Objectives: This study aimed to examine the associations among three types of cumulative disadvantages: long term poverty, spatial disadvantage, and multiple exclusions using a Cumulative dis/advantage (CDA) and life course perspective. Method: A sample of 419 Chinese adults aged 60 and older from three communities in Shanghai completed a structured questionnaire. Multiple exclusions were measured by variables related to material resources, housing conditions, social relations, civic activities, basic services, and neighbourhood factors. Hierarchical regression was implemented by SPSS 25 and moderation analysis was performed with the SPSS macro PROCESS from Hayes (2013). Results: 39% of respondents reported that they experienced multiple exclusions and one in five respondents report often or most time living in poverty. Regression analysis indicated that experience long-term poverty and length of living in the same neighbourhood is positively associated with multiple exclusions in later life and these associations are not attenuated by demographics, and health factors. But, moderation analysis showed the length of living in the same neighbourhood has significant moderating effect on the relationship between long term poverty and multiple exclusions, particularly for older adults living in the same neighbourhood for more than 30 years. Discussion: The study findings illustrate the need to consider not only life course risks such as long-term poverty but also spatial disadvantages in addressing multiple social exclusions among older Chinese adults.

